# Knowledge and attitudes of healthcare professionals working in a training and research hospital on early diagnosis of cervical cancer (a Somalia example): cross-sectional study

**DOI:** 10.1186/s12905-022-01808-9

**Published:** 2022-06-13

**Authors:** Şeyma Zehra Altunkurek, Samira Hassan Mohamed, Eda Şahin, Sümeyra Yilmaz

**Affiliations:** 1 Public Health Nursing Department, Gulhane Faculty of Nursing, University of Health Sciences, Ankara, Türkiye; 2 Somalia Mogadishu Recep Tayyip Erdoğan Faculty of Health Sciences, University of Health Sciences, Mogadishu, Somalia; 3 Obstetric and Gynecologic Nursing Department, Health Science Faculty, Giresun University, Giresun, Türkiye; 4 Public Health Nursing Department, Faculty of Health Science, University of Gazi, Ankara, Türkiye

**Keywords:** Cervical cancer, Prevention, Screening, Knowledge, Healthcare professionals

## Abstract

**Background:**

Despite the early diagnosis and treatment of cervical cancer, it is still a significant public health problem in Somalia. This study was conducted to evaluate the knowledge and attitudes of healthcare professionals towards the early diagnosis of cervical cancer.

**Methods:**

This study was conducted in Mogadishu, the capital of Somalia, between December 2020 and February 2021. The cross-sectional study consisted of a total of 280 healthcare professionals. The study data was collected using a questionnaire consisting of 38 questions evaluating the knowledge and attitudes of all healthcare professionals towards the early diagnosis of cervical cancer, an additional 15 questions for women only, and a total of 43 questions.

**Results:**

22.1% of the participants received cervical cancer training during vocational education and training. Cervical cancer education after graduation is 16.8%, and the rate of providing education to patients is only 29.6%. The rate of female healthcare professionals having a Pap smear test is 2%. The participants' cervical cancer total knowledge score was 16.5 ± 6.69, and the success rate was 63.46. The highest success rate in knowledge subgroup questions was HPV questions with 69.6. A statistically significant difference was found between the participants' profession, training on the subject during their vocational education, and total knowledge scores (*p* < 0.001). When the knowledge question subscales were compared, a significant difference was found between participants' gender and HPV questions subscale score (*p* = 0.028). A statistically significant difference was found between the participants' professions, receiving training on the subject during vocational training, and all subscales (*p* < 0.05). A statistically significant difference was found between the participants' income status and risk factors questions scores (*p* = 0.026).

**Conclusion:**

This study shows that the knowledge and training of healthcare professionals working in a training and research hospital in Somalia for early cervical cancer diagnosis are not sufficient. In addition, it reveals that female healthcare professionals have almost no Pap smears. Therefore, studies and training should be planned to train all healthcare professionals, especially female healthcare professionals, and overcome all possible obstacles to the acceptance of the screening tests by women.

## Introduction

Among the gender-specific cancers, gynecological cancers seen in women are an important public health problem in women's health, as they are familiar and frequently fatal diseases. Cervical cancer ranks second after breast cancer in women worldwide and ranks first among gynecological cancers [1]. Worldwide, approximately 266,000 women die from this disease each year, and 87% of these deaths occur in underdeveloped regions [2]. Therefore, it is one of the most common malignancies in developing countries and is the leading cause of cancer death in women. The highest incidence and death rates are seen in sub-Saharan Africa, Latin America, and South Asia [3]. In sub-Saharan Africa, 34.8 new cases of cervical cancer are diagnosed per 100,000 women annually, and 22.5 per 100,000 women die from the disease. This figure is higher than 6.6 and 2.5 per 100,000 women, respectively, in North America. Significant differences can be explained by low preventive health behavior and lack of access to effective screening services that facilitate early detection and treatment [4]. For example, 13.2 new cases of cervical cancer in Somalia are diagnosed per 100,000 women per year, and 10.2 per 100,000 women die from the disease [1].

Cervical cancer is a preventable disease with early diagnosis. One of the primary prevention methods is to raise awareness in society and ensure that healthcare professionals have sufficient correct information about prevention and screening methods [5]. The Organization for the Prevention of Cervical Cancers, collaborating with WHO, stated that all healthcare professionals should provide counseling and health training on the prevention of cervical cancer to women whenever and wherever they can [6]. The level of knowledge and awareness of healthcare professionals, who are responsible for enlightening society about cervical cancer prevention, screening, and early diagnosis, will increase the quality of service, and cervical cancer will be better combated. When the literature is examined, studies show that the level of knowledge of healthcare professionals about cervical cancer, early diagnosis, prevention, and screening is low, as well as studies showing that female healthcare professionals, who should be role models for society, have insufficient knowledge and attitudes towards cervical cancer, and reveal that screening rates are low. Studies show that it is not at the desired level [2, 3, 6–11].

Healthcare professionals have an important place in preventing cervical cancer, especially in African countries such as Somalia. Studies have been carried out in Africa on the knowledge, attitudes, and practices of cervical cancer prevention among healthcare professionals in reaching society in the prevention of cervical cancer [12–18], but this is the first study of healthcare professionals in Mogadishu, Somalia. This study provides valuable information for Somalia and other countries for planned steps to prevent cervical cancer in that region. This study aimed to determine the knowledge and attitudes of healthcare professionals working in Mogadishu, Somalia, Turkey Recep Tayyip Erdoğan Training and Research Hospital towards the early diagnosis of cervical cancer.

## Methods

### Study area, designed period

This cross-sectional study was conducted at Mogadishu Recep Tayyip Erdoğan Training and Research Hospital in Mogadishu, Somalia, between December 2020 and February 2021. Mogadishu is the capital of Somalia, and this training and research hospital is the most significant health facility in Mogadishu. There are 359 healthcare professionals employed in the hospital.

### Study population

The entire study population was formed by a random selection of male and female healthcare professionals working in Mogadishu Recep Tayyip Erdoğan Training and Research Hospital.

### Eligibility criteria

All male and female employees who were randomly selected from the healthcare professionals in the Training and Research Hospital and volunteered to participate in the study were included in the study.

### Sample size determination

The sample size for this study was determined using the single population ratio formula: the ratio of healthcare professionals with sufficient knowledge of cervical cancer is 50% (since there was no previous study in the study area). Level of significance 5% (α = 0.05), Z α/2 = 1.96 and margin of error 5% (d = 0.05). Adding a 10% non-response rate and multiplying by 2 (design effect), the total sample size required forth is study appeared to be 186.20% non-response rate was calculated as 224. However, 280 healthcare professionals who volunteered for the research were included in this study as a sample. 78% of the research population was reached.

### Variables

The dependent variables of this study were knowledge questions about cervical cancer. Independent variables consisted of socio-demographic characteristics and related subjects (age, marital status, educational status, occupation, income status, HPV testing for female employees, family history of cervical cancer, etc.).

The elemental form consisted of 12 questions containing the socio-demographic characteristics of the participants, created by the researchers by scanning the literature, 15 questions containing the HPV attitudes and practices of female employees [7–9], and 26 questions to measure cervical cancer knowledge level for healthcare professionals in Turkish. The questionnaire form was collected from the participants through a pre-tested, interviewer-administered questionnaire adapted and structured for this study. The questionnaire measured cervical cancer knowledge level, with 6 questions about cervical cancer general information and symptoms [1, 2, 6, 16, 17 and 20th statements], 9 questions about risk factors [3, 7–10], statements [13, 14, 18 and 25], 6 questions about early diagnosis and screening [statements 4, 5, 12, 15, 19 and 26], and 5 questions about HPV [11, 21, 22, 23 and 24 statements] and consisted of a total of 26 information questions. There are "true," "false," and "don't know" options for knowledge questions. Correct answers were scored as "1" and wrong answers as "0". The lowest score obtained from the questions is "0", while the highest score is 26 [10]. It was found that reliability, item discrimination, and item difficulty indices were not specified in the original study on the knowledge test. In this study, reliability, item discrimination, and item difficulty indices related to the knowledge test were calculated. Confidence coefficients Cronbach's Alpha 0.82; Split-Half (odd–even) Correlation 0.67; Spearman-Brown Prophecy 0.81; KR21 0.80; KR20 is calculated as 0.82. An item discrimination value higher than 0.20 [19] and an item difficulty index of 0.60 or higher [20] are considered sufficient for using the item. In this study, as a result of the analysis, item discrimination values of 26 items varied between 0.34 and 0.68; item difficulty values ranged from 0.64 to 0.82. The questionnaire was prepared in English and translated into the local language, Somali, and then into English to check for consistency. Data were collected during the data collection process with two researchers, one who is fluent in the Somali language and another researcher who is fluent in the English language.

### Data analysis

In evaluating the data was used by IBM Corp. Released 2013. IBM SPSS Statistics for Windows, Version 22.0. Armonk, NY: IBM Corp. Descriptive statistics defined socio-demographic characteristics (frequency, percentage, mean and standard deviation, minimum–maximum). To determine the distribution of the data, skewness and kurtosis coefficients, coefficient of variation, histogram, normal and detrend plots, the Kolmogrow Smirnow test was applied. In the evaluation, it was observed that the data were not normally distributed. The Mann–Whitney U test was used to compare independent paired groups, and the Kruskal Wallis analysis was used to reach more than two groups. The statistical significance limit was accepted as 0.05.

## Results

The mean age of the participants was 27.19 ± 3.44, 52.5% were female, most of them were single (52.1%) with a bachelor's degree (46.8%) and had a monthly average income (86.4%). Participants generally consisted of nurses (46.8%), and their average occupation duration was 4.38 ± 2.5. Those with a family history of cervical cancer are 4.3%, those who receive vocational training on cervical cancer 22.1%, and 16.8% receive training on this subject after graduation. Of the participants, 29.6% provided education to their patients about cervical cancer, and 40% recommended Pap smear testing (Table 1).

**Table 1:** Socio-demographic characteristics of healthcare professionals and their distribution regarding cervical cancer awareness status (n = 280)

Demographic features	n	%
Age	27.19 ± 3.44	
*Gender*		
Female	147	52.5
Male	133	47.5
*Marital status*		
Single	146	52.1
Married	126	45
Divorced	8	2.9
*Educational status*		
High school	6	2.1
Associate degree	39	13.9
Undergraduate	131	46.8
Postgraduate	104	37.2
*Monthly income status*		
Low	21	7.5
Middle	242	86.4
High	17	6.1
*Profession*		
Doctor	89	31.8
Nurse	131	46.8
Midwife	12	4.3
Health officer	16	5.7
Other	32	11.4
Occupation period	4.38 ± 2.51	
*Family history of cervical cancer*		
Yes	12	4.3
No	268	95.7
*Status of receiving cervical cancer training during vocational education*		
Yes	62	22.1
No	158	56.4
I do not remember	60	21.4
*Status of receiving cervical cancer education after graduation*		
Yes	47	16.8
No	197	70.4
I do not remember	36	12.8
*Providing education/information about cervical cancer to patients*		
Yes	83	29.6
No	197	70.4
*Recommending cervical cancer screening to their patients*		
Yes	112	40
No	168	60

22.4% of female participants had their first marriage between 17 and 23. Those who never married are in the majority (57.1%), and the mean age of first pregnancy is 23.26 ± 2.49. Those who have never been pregnant (66%) and never given birth (68.7%) are in the majority. In addition, 18.4% of female participants had a history of previous vaginal infection, and 1.4% had a sexually transmitted disease (Table 2).

**Table 2:** Some characteristics of female healthcare professionals regarding the obstetric history and cervical cancer (n = 147)

Features	n	%
*First marriage age*		
17–23	33	22.4
24–29	29	19.7
*Number of marriages*		
Never married	84	57.1
1	59	40.2
2	3	2
3	1	0.7
First gestational age	23.26 ± 2.49	
*Total number of pregnancies*		
0	97	66
1–3	38	25.9
Four and above	12	8.1
*Number of births*		
0	101	68.7
1–3	38	25.9
Four and above	8	5.4
*History of previous vaginal infection*		
Yes	27	18.4
No	120	81.6
*Status of having a sexually transmitted disease*		
Yes	2	1.4
No	145	98.6
*Use of oral contraceptives for more than five years*		
Yes	1	0.7
No	146	99.3
*Pap smear test status*		
Yes	3	2
No	144	98
*Last scan time*		
One year ago	1	33.3
Two years ago	2	66.7
Last scan location*		
Hospital	3	100
Scan frequency*		
Once a year	1	33.3
When there is a complaint	2	66.7
Reason for Pap test*		
For general health control purposes	1	33.3
Doctor's recommendation for complaints such as discharge, itching, bleeding, etc	2	66.7
Pap test result		
Normal Finding	1	33.3
Cervical Erosion	2	66.7
Reason for not having Pap test**		
No complaints, no need	27	45.8
Not being sexually active	2	3.4
Shame	4	6.8
Don't think about doing it in the future	6	10.2
Lack of knowledge	20	33.8

* These questions were directed to people who gave pap test (n = 3), ** This question was directed to people who didn't give pap test (n = 144)

None of the female participants smoke. In addition, 0.7% had been using oral contraceptives for more than five years, and 2% had previously had a Pap smear. They preferred the hospital as the screening site and the frequency of complaints (66.7%). The reason for having a swab test was on a doctor's recommendation due to complaints such as discharge, itching, bleeding, etc., at 66.7%. Those who did not have a swab test stated that they did not have any complaints and did not need it 45.8% (Table 2).

62.5% of the respondents stated that cervical cancer is a preventable disease, and 85% know that early diagnosis and treatment are possible. 38.6% of the participants believe that every woman is equally at risk to develop cervical cancer. Those who know that the Pap smear/HPV test is done for screening purposes are 65.7% and 57.1% think every sexually active woman should have it done (Table 3).

**Table 3:** Distribution of healthcare professionals' responses to cervical cancer information questions (n = 280)

Information statements	True		False		I don't know	
	n	%	n	%	n	%
1. Cervical cancer is a preventable disease	175	62.5	64	22.9	41	14.6
2. Early diagnosis and treatment of cervical cancer are possible	238	85	13	4.6	29	10.4
3. Every woman has equal risk of developing cervical cancer	108	3.6	118	42.1	54	19.3
4. Pap smear/HPV test is done for screening purposes	184	65.7	22	7.9	74	26.4
5. Every sexually active woman should have the Pap smear test done	160	57.1	50	17.9	70	25
6. Bleeding between menstrual cycles is an early sign of cervical cancer	123	43.9	73	26.1	84	30
7. Sexual experience at an early age (16 years and under) is a risk factor for cervical cancer	111	39.6	98	35	71	25.4
8. Smoking is one of the risk factors for cervical cancer	173	61.8	45	16.1	62	22.1
9. Polygamous sex life is a risk factor for cervical cancer	142	50.7	46	16.4	92	32.9
10. Having a family history of cancer does not increase the risk of cervical cancer	81	28.9	150	53.6	49	17.5
11. There is no protective vaccine against HPV (human papillomavirus)	88	31.4	112	40	80	28.6
12. Diagnosis of cervical cancer is made by Pap smear test	176	62.9	38	13.6	66	23.5
13. Pregnancy and delivery at an early age (under 18 years of age) are risk factors for cervical cancer	93	33.2	108	38.6	79	28.2
14. Sexually transmitted diseases are risk factors for cervical cancer	188	67.1	45	16.1	47	16.8
15. Pap smear/HPV test should be done only when there is a gynecological (gynecological) complaint	125	44.7	86	30.7	69	24.6
16. Increased vaginal discharge and color changes are early signs of cervical cancer	111	39.6	98	35	71	25.4
17. Bleeding and pain after sexual intercourse are not early signs of cervical cancer	90	32.1	111	39.6	79	28.3
18. The high number of births (3 or more) is a risk factor for cervical cancer	76	27.2	137	48.9	67	23.9
19. To prevent cervical cancer, it is necessary to regularly have a Pap smear/HPV test	149	53.3	69	24.6	62	22.1
20. Postmenopausal (post-menopausal) bleeding is one of the early symptoms of cervical cancer	166	59.3	43	15.4	71	25.3
21. HPV is a sexually transmitted virus	179	63.9	38	13.6	63	22.5
22. Cervical cancer and pre-cancerous cells are not associated with HPV	65	23.3	125	44.6	90	32.1
23. HPV causes genital warts	159	56.8	44	15.7	77	27.5
24. HPV infection is a risk factor for cervical cancer	163	58.2	28	10	89	31.8
25. Oral contraceptives (birth control pill) used for more than five years is not a risk factor for cervical cancer	89	31.8	105	37.5	86	30.7
26. Pap smear test can be done every five years if it is done together with HPV screening	119	42.5	37	13.2	124	44.3

The rate of participants who know that bleeding between periods is an early sign of cervical cancer is 43.9%. The rate of those who believe that sexual experience at an early age is a risk factor for cervical cancer is 39.6%. In addition, 50.7% of the participants know that polygamous sexual life is a risk factor for cervical cancer, and 31.4% think that there is no vaccine to protect against HPV. While 62.9% of the participants know that cervical cancer diagnosis is made with the Pap smear test, 38.6% do not think that pregnancy and delivery at an early age (under 18 years old) are risk factors for cervical cancer. Those who believe that sexually transmitted diseases are a risk factor for cervical cancer are 67.1%. Those who think that Pap smear/HPV test should only be done with a gynecological complaint are 44.7%.

Still 39.6% know that increased vaginal discharge and color changes are early signs of cervical cancer. Those who did not believe that bleeding and pain after sexual intercourse could be an early sign of cervical cancer were 32.1%. While the rate of those who do not know that a high number of births (3 or more) is a risk factor for cervical cancer is 48.9%, the rate of those who know that regular Pap Smear/HPV test is required to be protected from cervical cancer is 53.3%. The rate of those who know that postmenopausal bleeding is one of the early symptoms of cervical cancer is 59.3% and the rate of those who know that HPV is a sexually transmitted virus is 63.9%.

Those who think that cervical cancer and pre-cancerous cells are associated with HPV 44.6%, those who know that HPV causes genital warts are 56.8%, and those who know that HPV infection is a risk factor for cervical cancer 58.2%. While the majority of the participants (37.5%) know that oral contraceptive (birth control pill) used for more than five years is a risk factor for cervical cancer, they do not know that if the Pap smear test is performed together with HPV screening, it can be done every five years (44.3%).

The participants' knowledge questions mean scores are presented in Table 4. The highest success rate (69.6%) was seen in the HPV questions and the lowest success rate (57%) in screening questions. Therefore, the mean total knowledge score was determined as 16.5 ± 6.69 (63.46%).

**Table 4:** The mean scores of the knowledge questions of the healthcare professionals (n = 280)

Knowledge question subgroups	Min–Max	Mean ± SD	Success rate (%)
Scores from cervical cancer general knowledge and symptom questions	0–6	4.13 ± 1.83	68.83
Score from risk factors questions	0–9	5.46 ± 2.7	60.66
Score from screening questions	0–6	3.42 ± 1.96	57
Score from HPV questions	0–5	3.48 ± 2.19	69.6
Total knowledge score	0–26	16.5 ± 6.69	63.46

Gender and the score obtained from the HPV questions are significantly related, and men have a higher mean score than women (*p* = 0.028). Cervical cancer knowledge scores were found to be different according to the participants' professions. The average success rate of doctors in all fields is significantly higher than in other professions (*p* < 0.001). Those with low monthly income received higher scores on the risk factors questions (*p* = 0.026). Healthcare professionals who received training on the subject during vocational training had a significantly higher average score in all question groups than the others (Table 5).

**Table 5:** Comparison of cervical cancer knowledge scores according to some socio-demographic characteristics and awareness levels of healthcare professionals

Features	Score from general knowledge and symptom questions	Score from risk factors questions	Score from screening questions	Score from HPV questions	Total knowledge score
*Gender*					
Female	4.21 ± 1.95	5.23 ± 2.66	3.2 ± 2.06	3.21 ± 2.27	15.85 ± 7.02
Male	4.05 ± 1.69	5.71 ± 2.74	3.67 ± 1.82	3.78 ± 2.06	17.22 ± 6.24
	*p* = 0.352	*p* = 0.108	*p* = 0.05	*p* = 0.028	*p* = 0.130
*Profession*					
Doctor	4.67 ± 1.75	6.22 ± 2.67	4.35 ± 1.64	4.48 ± 2.11	19.74 ± 5.94
Nurse	3.85 ± 1.75	5.35 ± 2.73	3.06 ± 1.86	3.11 ± 1.95	15.38 ± 6.35
Midwife	4.41 ± 2.1	4.16 ± 1.64	2.83 ± 2.4	2.33 ± 2.18	13.75 ± 6.04
Health officer	4.37 ± 2.06	4.56 ± 2.27	2.93 ± 2.04	3.06 ± 2.35	14.93 ± 7.13
Other	3.56 ± 186	4.71 ± 2.73	2.78 ± 2.18	2.84 ± 2.37	13.9 ± 7.01
	*p* = 0.006	*p* = 0.004	*p* < 0.001	*p* < 0.001	*p* < 0.001
*Income status*					
Low	4.42 ± 2.33	6.9 ± 3.19	2.52 ± 1.77	3.38 ± 2.31	17.23 ± 8.01
Middle	4.17 ± 1.78	5.42 ± 2.62	3.54 ± 1.94	3.56 ± 2.16	16.71 ± 6.5
High	3.17 ± 1.55	4.17 ± 2.6	2.94 ± 2.16	2.41 ± 2.29	12.7 ± 6.1
	*p* = 0.054	*p* = 0.026	*p* = 0.061	*p* = 0.115	*p* = 0.052
*Family history of cancer*					
Yes	3.58 ± 1.72	5.33 ± 2.3	3.5 ± 1.78	2.58 ± 2.15	15 ± 6.74
No	4.16 ± 1.83	5.46 ± 2.72	3.42 ± 1.97	3.52 ± 2.19	16.57 ± 6.69
	*p* = 0.408	*p* = 0.943	*p* = 0.903	*p* = 0.152	*p* = 0.456
*Receiving training on the subject during vocational training*					
Yes	4.85 ± 1.73	6.24 ± 2.35	4.22 ± 1.96	4.32 ± 1.93	19.64 ± 5.74
No	3.89 ± 1.72	5.39 ± 2.72	3.17 ± 1.86	3.3 ± 2.25	15.77 ± 6.51
I do not remember	4.01 ± 2.03	4.83 ± 2.85	3.36 ± 2.04	3.08 ± 2.1	15.2 ± 7.16
	*p* = 0.002	*p* = 0.008	*p* = 0.001	*p* = 0.002	*p* < 0.001
*Status of providing education to patients on the subject*					
Yes	4.28 ± 1.64	5.84 ± 2.35	3.81 ± 1.81	3.69 ± 1.96	17.65 ± 6.02
No	4.07 ± 1.9	5.29 ± 2.83	3.26 ± 2	3.39 ± 2.28	16.02 ± 6.9
	*p* = 0.177	*p* = 0.077	*p* = 0.054	*p* = 0.297	*p* = 0.061

## Discussion

This study was cross-sectional with 280 people to determine the knowledge and attitudes of healthcare professionals working in a training and research hospital in Mogadishu, Somalia, towards the early diagnosis of cervical cancer.

In our study, when the training status of the healthcare professionals on cervical cancer was examined, only 22.1% of the participants stated that they received training on cervical cancer during their vocational training. When receiving postgraduate education is evaluated, a meager rate of 16.8% noted that the participants received in-service training after graduation, and the rest stated that they did not receive or do not remember. In contrast with this study, in a similar study conducted in Turkey, approximately 3/4 of the healthcare professionals stated that they received training on cervical cancer during their vocational training, and when the postgraduate education status was evaluated 2/3 of the participants showed that they received in-service training after graduation [10]. Our study shows that the rate of those who received training on cervical cancer during vocational training and after graduation is meager and insufficient. For this reason, it is essential to inform healthcare professionals, refresh their knowledge, and keep them informed of developments through vocational training to protect both their own health and public health.

Almost all female healthcare professionals (98%) in our study had not been screened for cervical cancer before. Other studies in the literature have obtained similar results to our study; they showed that female healthcare professionals had cervical cancer screening at a low rate. The rate of having them varied between 6 and 41% [13, 15, 18, 21–25]. In our study, female healthcare professionals who did not undergo screening stated that they did not have any complaints and therefore did not need and did not have knowledge. On the other hand, other studies have stated the reasons for not participating in the screening, such as lack of access to screening services, false beliefs about cervical cancer and screening, and fear of the consequences for female healthcare professionals. These results may suggest that the participation of female healthcare professionals in cervical-screening programs is not at the desired level and that there is a gap in practice and training on this issue.

When the status of the participants was to inform and recommend their patients about cervical cancer, 29.6% stated that they gave education/information about cervical cancer to their patients, and 40% indicated that they recommended screening for cervical cancer to their patients. Contrary to our study, almost all the participants in the Revolution study stated that they gave education/information about cervical cancer to their patients and offered screening for cervical cancer to their patients [10]. In a study conducted by Kaya et al. in 2014 with primary care and tertiary healthcare professionals, the rate of those recommending HPV-DNA screening tests to the target group was 28.17%, while the rate of recommending Pap smear tests was found to be 69.77% [26]. According to this study, we think that the low level of cervical education and screening recommendation given to our patients is due to the lack of awareness and education of the healthcare professionals.

In our study, the mean score of healthcare professionals from information statements was 16.5 ± 6.69. Considering that the maximum score that can be obtained is "26", it was determined that the scores of the healthcare professionals are close to the middle level. In a study conducted with healthcare professionals in Uganda, the level of knowledge (60%) of the participants about cervical cancer was similar to our study [17]. In the study conducted by Devrim (2019) with primary healthcare professionals, it was determined that the average score of the participants from the cervical information questions was 18.99 ± 4.50, and the information was slightly higher than our study [10]. When compared with the study conducted with healthcare professionals in the Ivory Coast, it was seen that the cervical knowledge score of the participants (55.7%) was lower than our study [21]. However, other studies that were Ethiopia (86.9%) [15], India (85%) [27], Nigeria (98.6%) [28], Pakistan (%88.1) [29] and Burundi's (76.3%) was found higher than our study [30]. It was found that the cervical score averages of the studies carried out were higher.

When the knowledge question subgroups were examined in this study, the most successful knowledge statement group was the HPV questions group, with a success rate of 69.6%. However, in different studies conducted with healthcare professionals, it was determined that the average score of the screening questions is relatively high, with a success rate of 83–84% [10]. In a study conducted with nurses in India, 79% of the participants knew about cervical cancer screening methods, and 91% knew about the HPV vaccine. Again, the same study showed that 82% were aware of Pap smear, and 89% had a positive attitude towards it [31]. However, in a study conducted in Tanzania to determine cervical cancer and cervical screening practices, less than half of the nurses had sufficient knowledge about cervical cancer [18]. The results in our study and the literature may suggest that the understanding of cervical cancer is not at the desired level among healthcare professionals.

In our study, in the comparison of the socio-demographic characteristics of the healthcare professionals and the mean total score of cervical cancer knowledge, it was observed that the knowledge scores were not affected by gender, marital status, or monthly income, but were affected by the training and occupational groups during vocational training. In the study conducted with healthcare professionals working in primary health care institutions in Turkey, results similar to our research were obtained; it was stated that cervical cancer knowledge score averages were affected by occupational groups and education levels [10]. The literature shows that studies on cervical cancer information mainly apply to nurses and female healthcare professionals [12, 15, 18, 31]. However, choosing the cervical cancer information of all healthcare professionals, especially in Africa where the incidence and prevalence of cervical cancer are high, may suggest that it is essential for training needs.

In our study, a significant relationship was found between the sexes of healthcare professionals and the HPV question subgroups of the screening questions. While men scored high in all knowledge questions, only these two subgroups showed a significant difference. However, in the study conducted by Batkın with healthcare professionals, no meaningful relationship was found between gender and question averages. The difference in this study suggests that male healthcare professionals are doctors, and therefore their knowledge level is higher than other workers.

In our study, in the comparison between the cervix information subgroup scores of healthcare professionals and occupational groups, in all score types, it was found that the doctors were not in agreement with other similar studies [10, 12–15]. While a significant difference was found in our research, in a similar study conducted in Turkey, it was stated that although physicians had higher scores compared to other occupational groups, it was not significant. The results suggest that there is a need for training of non-doctor healthcare professionals about cervical cancer information and early diagnosis screening.

In order for community-based screening programs to be successful, the community should know about HPV infection and cervical cancer. In order to raise the awareness of society on this issue, it is particular crucial that healthcare professionals have knowledge of this issue [32].

In our study, it was observed that there was a significant relationship between the cervical knowledge question subgroup scores of those who received training on cervical cancer during vocational training. Healthcare professionals who received training had higher scores. Similar to the finding in our study, in a study conducted with healthcare professionals in Turkey, the rate of knowing the cervical cancer screening program correctly was found to be higher in those who had previously received training on this subject [33]. In addition to these results, there are also experimental studies showing that the level of cervical cancer knowledge increases with the training given in the literature [34, 35]. According to these findings, it is thought that it is vital that all healthcare professionals who are involved in providing services to the public in the early diagnosis and treatment of cancer are both informed about the subject during their vocational training and supported by in-service training.

## Limitations

The aim of our study is to reveal the knowledge and attitudes of healthcare professionals working in a research hospital toward the early diagnosis of cervical cancer. Therefore, the findings are valid only for the sample to which it was applied. It cannot be generalized to the entire population.

## Conclusion

According to the results of this study, it was determined that the knowledge of the healthcare professionals working in a training and research hospital in Somalia about the early diagnosis of cervical cancer is not sufficient. It was observed that there is a more significant lack of information, especially about screening programs. It was determined that health professionals who were except doctors and those who did not receive information during occupational training has less knowledge about cervical cancer. It was determined that those who did not receive information had less knowledge about cervical cancer. It was determined that the rate of female healthcare professionals having a Pap smear test is almost nonexistent. These results show that healthcare professionals need training programs to explain the importance of early diagnosis to society in the prevention of cervical cancer and to increase participation. In addition, it is necessary to ensure that women, especially female healthcare professionals, participate in programs about applications for cervical cancer early diagnosis methods.

## Data Availability

The datasets generated and analyzed during the current study are not publicly available due to different languages but are available from the corresponding author on reasonable request.

## Abbreviations

IARC: The International Agency for Research on Cancer
WHO: The World Health Organization
HPV: Human Papilla Virus
